# Comparative Evaluation of Indirect Immunofluorescence and NS-1-Based ELISA to Determine Zika Virus-Specific IgM

**DOI:** 10.3390/v10070379

**Published:** 2018-07-19

**Authors:** Fernando De Ory, María Paz Sánchez-Seco, Ana Vázquez, María Dolores Montero, Elena Sulleiro, Miguel J. Martínez, Lurdes Matas, Francisco J. Merino, Working Group for the Study of Zika Virus Infections

**Affiliations:** 1 Laboratorio de Serología y Arbovirus, Centro Nacional de Microbiología, Instituto de Salud Carlos III, Majadahonda 28220, Spain; paz.sanchez@isciii.es (M.P.S.-S.); a.vazquez@isciii.es (A.V.); 2 Centro de Investigación Biomédica en Red en Epidemiología y Salud Pública, Instituto de Salud Carlos III, Madrid 28029, Spain; lmatas.germanstrias@gencat.cat; 3 Hospital Universitario La Paz, Madrid 28046, Spain; mdolores.montero@salud.madrid.org; 4 Hospital Vall d’Hebron, Barcelona 08035, Spain; esulleir@vhebron.net; 5 Hospital Clínic, Barcelona 08036, Spain; myoldi@clinic.cat; 6 Hospital Universitari Germans Trias i Pujol, Badalona 08916, Spain; 7 Hospital Severo Ochoa, Leganés 28911, Spain; franciscojesus.merino@salud.madrid.org

**Keywords:** Zika virus, dengue viruses, flavivirus, ELISA, indirect immunofluorescence, plaque reduction neutralization test, polymerase chain reaction, cross-reactions

## Abstract

Differential diagnosis of the Zika virus (ZIKV) is hampered by cross-reactivity with other flaviviruses, mainly dengue viruses. The aim of this study was to compare two commercial methods for detecting ZIKV immunoglobulin M (IgM), an indirect immunofluorescence (IIF) and an enzyme immunoassay (ELISA), using the non-structural (NS) 1 protein as an antigen, both from EuroImmun, Germany. In total, 255 serum samples were analyzed, 203 of which showed laboratory markers of ZIKV infections (PCR-positive in serum and/or in urine and/or positive or indeterminate specific IgM). When tested with IIF, 163 samples were IgM-positive, while 13 samples were indeterminate and 78 were negative. When IIF-positive samples were tested using ELISA, we found 61 positive results, 14 indeterminate results, and 88 negative results. Among the indeterminate cases tested with IIF, ELISA analysis found two positive, two indeterminate, and nine negative results. Finally, 74 of the 78 IIF-negative samples proved also to be negative using ELISA. For the calculations, all indeterminate results were considered to be positive. The agreement, sensitivity, and specificity between ELISA and IIF were 60.2%, 44.9%, and 94.9%, respectively. Overall, 101 samples showed discrepant results; these samples were finally classified on the basis of other ZIKV diagnostic approaches (PCR-positive in serum and/or in urine, IgG determinations using IIF or ELISA, and ZIKV Plaque Reduction Neutralization test—positive), when available. A final classification of 228 samples was possible; 126 of them were positive and 102 were negative. The corresponding values of agreement, sensitivity, and specificity of IIF were 86.0%, 96.8%, and 72.5%, respectively. The corresponding figures for ELISA were 81.1%, 65.9%, and 100%, respectively. The ELISA and IIF methods are both adequate approaches for detecting ZIKV-specific IgM. However, considering their respective weaknesses (low sensitivity in ELISA and low specificity in IIF), serological results must be considered jointly with other laboratory results.

## 1. Introduction

The Zika virus (ZIKV) is a mosquito-transmitted virus belonging to the flavivirus genus. This genus includes other human pathogens, such as the dengue virus (DENV), the West Nile virus, and the yellow fever virus. ZIKV shares some clinical and microbiological characteristics with DENV, making its diagnosis difficult.

Firstly, both viruses cause an exanthematic febrile disease, characterized by the presence of arthralgia and retroocular pain, although there are differential signs or symptoms, such as rash with pruritus, conjunctivitis, and limb edema (characteristic of ZIKV disease), or leucopenia/thrombocytopenia (characteristic of dengue) [1,2]. Secondly, they share a vector (mosquitoes of the genus, *Aedes*) and, consequently, a distribution area [3]. Thirdly, both viruses, as well as other flaviviruses, share antigenic reactivity, resolved in serological cross-reactivity when measuring specific antibodies [4]. For these reasons, it is important to have specific serological assays that can facilitate an adequate differential diagnosis.

Indirect immunofluorescence (IIF) is widely used to detect ZIKV antibodies, using ZIKV-infected cells to identify class-specific antibodies. However, using this approach, the high degree of cross-reactivity between ZIKV and other flaviviruses makes correct serological diagnosis difficult. The ZIKV non-structural (NS) 1 protein was identified as being largely specific to the virus [5], and, as a consequence, new ELISA assays were developed. Although some studies evaluated the use of ELISA reagents for determining immunoglobulin M (IgM) against ZIKV [6,7,8,9,10,11,12], no comparisons with IIF are available.

The aim of this study was to evaluate the comparative performance characteristics of an NS1 antigen-based ELISA and an IIF assay for identifying ZIKV-specific IgM. For this purpose we used a large panel of samples that were well characterized by molecular and serological approaches, including a real-time molecular assay, IgM and IgG ELISA, and the plaque reduction neutralization test (PRNT).

## 2. Materials and Methods

### 2.1. Samples

A total of 255 serum samples received in our laboratory were included in the study. Of these, 239 samples showed markers of ZIKV infection, including 203 cases from 201 adults (66 men, 135 women (30 pregnant)) and two newborns. The samples were received over a period of 18 months (1 January 2016–31 July 2017). The study was approved by the Ethical Committee of the Institute of Health Carlos III (code: CEI PI 64_2018). All patients had recently traveled to one or more Latin American countries. The samples were grouped as described below.

1. Seventy-one paired samples from 35 cases (three samples were available from one case):
  (a) Four cases (eight samples) were PCR-positive (using real-time PCR) in serum and urine, and ZIKV IgM-positive using IIF (five samples).
  (b) Five cases (10 samples) had a positive result with PCR in serum (one of them was negative in urine), and two samples from two cases were ZIKV IgM-positive using IIF.
  (c) Nine cases (19 samples) were PCR-positive only in urine (six were negative in serum), and ZIKV IgM-positive in eight (eight positive and four indeterminate samples).
  (d) The remaining 17 cases (34 samples; three with a PCR-negative result in serum and one in urine) were IgM-positive or indeterminate in at least one sample. Seven of them were ZIKV IgM-positive in acute and convalescent samples. Three cases showed seroconversion of ZIKV IgM. Of these, both samples of one case were indeterminate, and in the other case, the acute sample was indeterminate and the convalescent sample was positive. Finally, five cases (indeterminate to positive (two cases), positive to indeterminate (one case), positive to negative (one case), and indeterminate to negative (one case)), showed the presence of DENV IgM (four cases) or DENV IgG seroconversion (one case).
2. One hundred and sixty-eight samples:
  (a) Twenty-three samples were ZIKV IgM-positive, of which PCR was positive in serum and urine (one case), in serum only (eight cases), or in urine only (14 cases).
  (b) One hundred and one samples gave a positive result for ZIKV IgM; PCR was carried out in serum and urine in 28 of them, all of which gave a negative result.
  (c) Forty-four samples were negative for ZIKV IgM using IIF, with a positive PCR result in serum and urine (six cases), in serum only (15 cases), or in urine only (23 cases).

The remaining 16 samples came from recent dengue infections (nine samples from eight patients), as diagnosed by IgM detection, or past dengue infections (seven samples), as confirmed by IgG detection in the absence of IgM. These samples were taken during 2012, before the ZIKV epidemic in the Americas. The patients reported recent travel to Latin American countries. When tested for ZIKV IgM using IIF, two samples were positive, another two were indeterminate, and one other gave an uninterpretable result, with a strong background and no specific fluorescence.

### 2.2. Methods

ZIKV-specific IgM and IgG were tested using IIF with commercial reagents (Arbovirus Fever Mosaic 2, catalog #FR2668-1010-1G and #FR2668-1010-1M for IgG and IgM, respectively, obtained from EuroImmun, Lübeck, Germany). For testing IgM, samples were pretreated with an anti-human IgG (RF Absorbent, reference OUCG, Siemens, Marburg, Germany), to remove the interference due to the presence of rheumatoid factor and specific IgG to avoid false-positive results. Briefly, samples pretreated with a ten-fold dilution were incubated for 30 min at 37 °C in wells of slides containing ZIKV-infected cells. After washing with phosphate-buffered saline (PBS), pH 7.2, supplemented with Tween 20 (PBS-Tween), an anti-human IgM (goat)-fluorescein isothiocyanate conjugate was added to the wells and incubated as before. After the final washing, the slides were mounted in glycerol buffered with PBS, pH 8.4, and read in an inverted fluorescence microscope (Zeiss Axiovert 25, Jena, Germany) equipped with a mercury vapor lamp HBO50 (Osram, Munich, Germany), at a magnification of 200×. Samples showing fine-to-coarse granular cytoplasmic structures and/or net-like fluorescence with a dense perinuclear reactivity were considered to be positive. The kit included positive and negative controls. The results obtained using IIF were read by two different people. Samples where interpretations differed between the two assessors were considered to be indeterminate.

Dengue virus serology was done using commercial ELISA tests. Immunoglobulin M was determined with the Panbio Dengue IgM Capture ELISA, catalog #01PE20, while DENV IgG was determined with the Panbio Dengue IgG Indirect ELISA, catalog #01PE30, both obtained from Standard Diagnostics Inc., Geonggi-do, South Korea. Dengue virus assays use DENV 1–4 antigens, recombinant in the case of the IgM assay. Dengue virus IgM was confirmed using a background assay, as described elsewhere [13]. The ZIKV PRNT was assessed with an in-house test using Vero E6 cells and 100 tissue infective infectious dose 50% of ZIKV (African strain MR-766, isolated from a rhesus monkey in the Zika Forest, Uganda, in 1947, and maintained in our laboratory in Vero E6 cells). For neutralizing antibodies, samples were tested following titration in two-fold dilutions from 1/8. Samples were considered positive if neutralization of viral growth at a dilution greater than 128-fold was observed; samples with titers between eight-fold and 128-fold were considered indeterminate, while samples with a titer less than eight-fold were considered negative. Zika virus RNA was determined using a commercial assay of real-time PCR (RealStar^®^Zika Virus, altona Diagnostics, Hamburg, Germany, catalog number: 774AD-591013) [14].

The assay under evaluation was an ELISA using the NS1 protein (recombinant) from the virus (ELISA Zika virus IgM, catalog number EI 2668-9601 M, EuroImmun) as the antigen. Briefly, samples diluted 100-fold in a buffer containing anti-human IgG (goat) were incubated with the immobilized antigen NS1 for 1 h at 37 °C. The plate was washed three times, and an anti-human IgM (goat) peroxidase conjugate was added. After incubating for 30 min at room temperature and washing as before, a solution of 3, 3’, 5, 5’ tetramethylbenzidine/H_2_O_2_ was added as the enzyme substrate. The reaction was stopped with 0.5 M H_2_SO_4_, and the plate was read at 450 nm. A calibrator containing ZIKV-specific IgM included in the kit was tested in each run to define the cut-off of the test. Samples with absorbance/cut-off values greater than 1.1 were considered positive, and values less than 0.8 were considered negative. All other values were regarded as indeterminate.

All tests using commercial reagents were done strictly following the manufacturer’s instructions.

To establish the comparison, all indeterminate results using either IIF or ELISA were considered as positive.

## 3. Results

Overall, in the IIF assay, 163 samples were ZIKV IgM-positive, 13 were indeterminate, and 78 were negative. The result from the remaining sample could not be interpreted, and was excluded from the analysis.

The general comparison between IIF and ELISA for detecting ZIKV-specific IgM is illustrated in Table 1. Excluding the sample with an uninterpretable result, the ELISA test showed 67 samples to be positive, 16 to be indeterminate, and 171 to be negative.

Out of 163 positive IIF samples, 61 were positive, 14 were indeterminate, and 88 were negative with ELISA. Of the 13 indeterminate IIF samples, two were positive, two were indeterminate, and nine were negative with ELISA. Finally, 74 of the 78 IIF-negative samples were also negative with ELISA, while four were positive (Table 1).

Considering all indeterminate results to be positive, the comparison of ELISA and IIF provided values of agreement of 60.2% (95% confidence intervals (CIs): 53.9–66.2%; 153/254), a sensitivity of 44.9% (95% CIs: 37.5–52.5%; 79/176), and a specificity of 94.9% (95% CIs: 86.7–98.3%; 74/78).

All positive and indeterminate results with both IIF and ELISA were considered as true positives, and the samples showing a negative result with both assays were considered as negative. One hundred and one samples showed discrepant results with the two assays (listed in Table 2, Table 3 and Table 4). The samples were finally classified considering other specific ZIKV diagnostic approaches (PCR-positive in serum and/or in urine, IgG determinations using IIF or ELISA, and ZIKV PRNT-positive), when available.

Firstly, 28 discrepant samples, all with a positive (25 samples) or indeterminate (3 samples) result using IIF were finally classified as negative (Table 2). Ten of them (#1 to #10, Table 2) came from dengue cases, since they showed either DENV IgM and NS1 antigens (samples #1, #2, and #4), or DENV IgM in a follow-up sample (#3), or DENV IgM with ZIKV PRNT-negative and ZIKV PCR-negative in serum and urine (#5) or in serum only (#6), or DENV IgM in samples taken in 2012, before the outbreak of ZIKV in the Americas in 2015 (four samples, #7 to #10). Two additional cases tested negative for ZIKV PCR in urine and serum, and for ZIKV PRNT (#11, #12), while three more were negative for ZIKV PCR in serum (#13 and #14) or in urine (#15), and the remaining 13 samples (#16 to #28) were negative for PRNT.

Secondly, 47 samples with discrepant results were finally classified as positive (Table 3). Of these, 43 were positive (37 samples) or indeterminate (6 samples) using IIF and negative using ELISA, and four were positive using ELISA and negative using IIF. Of the samples giving a positive or indeterminate result using IIF, 12 were paired samples (#29 to #40, Table 3) from six cases that were positive (eight samples, #29, #30, #32, #33, #36, #37, #38, and #39) or indeterminate (four samples, #31, #34, #35, and #40) using IIF. Of these, four cases gave a positive result for ZIKV PCR three cases in urine (with a positive PRNT assay result in two of them) and one in serum. Two other paired samples were ZIKV PRNT-positive in acute and convalescent samples. Sample #41 was an acute sample whose corresponding convalescent sample showed IgG seroconversion with both ELISA and IIF. Samples #42 to #48 were convalescent ones, showing either a ZIKV PCR-positive result in urine and an PRNT-positive result (#42), or a positive or indeterminate result using IIF and/or ELISA in the corresponding acute samples (#43, #44, and #45), or seroconversion of IgM (#46) or IgG (#47 and #48). Sample #49 was an acute sample with a ZIKV PCR-positive result in urine. Samples #50 to #71 were single samples that were ZIKV PCR-positive in urine and serum (#50), ZIKV PCR-positive in serum and ZIKV PRNT-positive (#51), ZIKV PCR-positive in serum (#52, #53, and #54), ZIKV PCR-positive in urine and PRNT-positive (#55), and ZIKV PCR-positive in urine (#56, #57, #58, and #59). Samples #60 to #71 were single samples for which a single positive result in the ZIKV PRNT assay was demonstrated.

Of the four samples showing a positive result using ELISA but a negative one using IIF, two were convalescent samples showing a ZIKV PCR-positive result in urine and IgG seroconversion (#72), or an IgM-positive result using ELISA and an indeterminate result using IIF in the acute sample (#73). The latter samples were two single samples from cases that were ZIKV PCR-positive in urine (#74 and #75).

Finally, the other 26 samples showing discrepant results were considered unclassifiable, because not enough results were available (Table 4).

Thus, a final classification was possible for 228 samples (126 positive and 102 negative; Table 5). Indirect immunofluorescence showed 86% agreement (95% CIs: 80.6–90.1%; 196/228), 96.8% sensitivity (95% CIs: 91.6–99.0%; 122/126), and 72.5% specificity (95% CIs: 62.7–80.7%; 74/102). ELISA, on the other hand, showed 81.1% agreement (95% CIs: 75.3–85.9%; 185/228), 65.9% sensitivity (95% CIs: 56.8–73.9%; 83/126), and 100% specificity (95% CIs: 95.5–100%; 102/102).

## 4. Discussion

The best definition of a clinical case of ZIKV in areas with concurrent circulation of other flaviviruses is the presence of a rash with pruritus or conjunctival hyperemia, in the absence of any other general clinical manifestations [2]. However, there are difficulties in the differential diagnosis of individual cases with other arboviruses, especially DENV, due to the cross-reactions between the members of the flavivirus genus, and the chikungunya virus. Thus, the main problem in comparatively evaluating serological assays for ZIKV assays is the correct classification of cases. In the presented study, samples giving the same result with the compared methods were considered as true, as was the case in 153 samples (60.2%) of those analyzed, having obtained 101 discrepant results using IIF and ELISA. For the final classification of the discrepant results, the results of other infection markers (PCR-positive in urine and serum and/or IgG seroconversion and/or PRNT-positive for ZIKV, and IgM and/or IgG seroconversion and/or NS1 antigen detection for DENV) were taken into consideration when they were available. In this way, discrepant samples were finally classified as negative (28 samples; Table 3) or positive (47 samples; Table 4), while a definitive classification of the remaining 26 samples was not possible (Table 5). After the final classification of the discrepancies, the NS1-based ELISA showed excellent specificity when applied to samples that were received by the laboratory for the diagnosis of ZIKV infection. The high specificity obtained was consistent with that previously reported for the ELISA assay evaluated here [6,7,8,9], as well as for other tests based on the NS1 protein [9,10]. It is of particular note that the specificity of ELISA is markedly higher than that of IIF (Table 5).

The sensitivity, on the other hand, was lower using ELISA than with the IIF test. The figure obtained here for ELISA (65.9%) was better than those previously reported in the literature: 20.7% [10]; 32% [9]; 54% [8], and 58.8% [7]. The differences in sensitivity could be related to the different approaches used to establish the comparisons in the various reports. The lower sensitivity of ELISA in relation to IIF could be explained by the fact that only one protein (NS1) acted as an antigen in ELISA in contrast to the whole virus in IIF, or by the kinetics of the antibodies against NS1, which could be delayed in relation to the antibodies detected against the complete virus, as detected in the IIF assay. In order to assess this aspect, the dates of bleeding of the paired samples showing discrepant results, and those which were finally classified as positive (samples #29 to #40, Table 3) were reviewed. Follow-up samples were taken between 12 days and 180 days after the first sample was taken, and in none of the six cases was the follow-up sample positive using ELISA. Therefore, it does not seem likely that the lower sensitivity of ELISA compared with that of IIF is related to a deferred response of the ZIKV IgG to the NS1 antigen.

The cross-reactivity between ZIKV and DENV, as well as that with other flaviviruses, as consistently reported [6,12], is of particular note. This issue is of particular interest, since patients with prior dengue infections were reported to not develop an IgM response against ZIKV NS1 [15]. Of the cases analyzed with a unique IIF-positive result for ZIKV IgM, 12 showed markers of recent DENV infection, either in samples that were classified as ZIKV-negative (#1 to #10, Table 2) or that were unclassifiable (#100 and #101, Table 4). All cases produced by DENV, included in Table 2, were confirmed as false-positive in IIF, since they showed markers of DENV infection, by IgM (all cases) and, more importantly, by detection of the NS1 antigen of DENV (samples #1, #2, and #4), in the absence of other markers of ZIKV infection. Also of interest are the IIF-positive results in the samples showing DENV IgM taken before the epidemic of the virus in the Americas (samples #7 to #10, Table 2). Nevertheless, ZIKV and DENV double infections cannot be completely ruled out; this seems to be the case for samples #100 and #101 (Table 4), which were considered as unclassifiable. Thus, the ELISA technique was confirmed as being more specific than IIF, and appeared not to be affected by the cross-reaction when analyzing cases of DENV infection, even in the presence of the DENV NS1 antigen. The specificity of the IIF and ELISA assays should be the subject of a broader study, including not only cases of DENV and ZIKV, but also, at least, cases of yellow fever.

In conclusion, the ELISA and IIF methods could both be adequate approaches for detecting ZIKV-specific IgM. However, considering their respective weaknesses (sensitivity in ELISA and specificity in IIF), serological results must be considered in the context of clinical and epidemiological data, and of other laboratory results.

## Figures and Tables

**Table 1:** Results of ELISA compared with indirect immunofluorescence (IIF).

		Indirect Immunofluorescence			
		Positive	Indeterminate	Negative	Uninterpretable
**ELISA**	**Positive**	61	2	4	0
	**Indeterminate**	14	2	0	0
	**Negative**	88	9	74	1

**Table 2:** Discrepant results finally classified as negative.

Number	ZIKV IgM		Other Results *	Final Classification
	IIF	ELISA		
**#1**	POS	NEG	Acute sample. DENV (NS1 Ag & IgM)	Negative (dengue)
**#2**	IND	NEG	Acute sample. DENV (NS1 Ag & IgM). PRNT ind (9 dao)	Negative (dengue)
**#3**	POS	NEG	Follow-up sample. DENV (IgM). PRNT neg (45 dao)	Negative
**#4**	POS	NEG	Single sample. DENV (NS1 Ag & IgM). PRNT neg	Negative (dengue)
**#5**	POS	NEG	Single sample. PCR neg (serum & urine), PRNT neg, DENV (IgM)	Negative (dengue)
**#6**	POS	NEG	Single sample. PCR neg (serum), PRNT neg, DENV (IgM)	Negative (dengue)
**#7–#9**	IND	NEG	Single sample. DENV (IgM). Sample from 2012	Negative (dengue)
**#10**	POS	NEG	Acute sample. DENV (IgM). Sample from 2012	Negative (dengue)
**#11, #12**	POS	NEG	Single sample. PCR neg (serum & urine), PRNT neg	Negative
**#13, #14**	POS	NEG	Single sample. PCR neg (serum)	Negative
**#15**	POS	NEG	Single sample. PCR neg (urine)	Negative
**#16–#28**	POS	NEG	Single sample. PRNT neg	Negative

* dao: days after onset; PRNT: plaque reduction neutralization test; ind: indeterminate result; pos: positive; neg: negative; ZIKV: Zika virus; DENV: dengue virus; NS1 Ag: non-structural protein 1 antigen; IgM: immunoglobulin M.

**Table 3:** Discrepant results finally classified as positive.

Number	ZIKV IgM		Other Results *	Final Classification
	IIF	ELISA		
**#29**	POS	NEG	Acute sample. PCR pos (serum) (1 dao)	Positive
**#30**	POS	NEG	Follow-up sample (180 dao)	Positive
**#31**	IND	NEG	Acute sample. PCR pos (urine) (n dao)	Positive
**#32**	POS	NEG	Follow-up sample. PRNT pos (n+18 dao)	Positive
**#33**	POS	NEG	Acute sample. PCR pos (urine) (n dao)	Positive
**#34**	IND	NEG	Follow-up sample. PRNT pos (n+25 dao)	Positive
**#35**	IND	NEG	Acute sample. PCR pos (urine) (7 dao)	Positive
**#36**	POS	NEG	Follow-up sample. PRNT ind (33 dao)	Positive
**#37**	POS	NEG	Acute sample. PRNT pos (n dao)	Positive
**#38**	POS	NEG	Follow-up sample. PRNT pos (n+12 dao)	Positive
**#39**	POS	NEG	Acute sample. PRNT pos (50 dao)	Positive
**#40**	IND	NEG	Follow-up sample. PRNT pos (78 dao)	Positive
**#41**	POS	NEG	Acute sample. IgG SC (IIF & ELISA)	Positive
**#42**	POS	NEG	Convalescent sample. PRNT pos. PCR pos (urine)	Positive
**#43**	POS	NEG	Convalescent sample. PCR neg (serum). Acute sample: IgM pos (IIF & ELISA)	Positive
**#44**	IND	NEG	Convalescent sample. Acute sample: IgM ind (ELISA)	Positive
**#45**	POS	NEG	Convalescent sample. Acute sample: IgM ind (IIF); pos (ELISA)	Positive
**#46**	POS	NEG	Convalescent sample. IgM SC (IIF). PRNT pos. Acute sample: PCR pos (serum & urine)	Positive
**#47, #48**	POS	NEG	Convalescent sample. IgG SC (ELISA)	Positive
**#49**	IND	NEG	Acute sample. PCR pos (urine)	Positive
**#50**	POS	NEG	Single sample. PCR pos (urine & serum)	Positive
**#51**	POS	NEG	Single sample. PCR pos (serum). PRNT pos	Positive
**#52–#54**	POS	NEG	Single sample. PCR pos (serum)	Positive
**#55**	POS	NEG	Single sample. PCR pos (urine). PRNT pos	Positive
**#56–#59**	POS	NEG	Single sample. PCR pos (urine)	Positive
**#60–#71**	POS	NEG	Single sample. PRNT pos	Positive
**#72**	NEG	POS	Convalescent sample. IgG SC (IIF & ELISA). PCR pos (urine)	Positive
**#73**	NEG	POS	Convalescent sample. Acute sample: IgM ind (IIF); pos (ELISA)	Positive
**#74, #75**	NEG	POS	Single sample. PCR pos (urine)	Positive

* SC: seroconversion.

**Table 4:** Unclassifiable discrepant results.

Number	ZIKV IgM		Other Results	Final Classification
	IIF	ELISA		
**#76**	POS	NEG	Acute sample. PRNT ind	Unclassifiable
**#77**	POS	NEG	Follow-up sample. PRNT ind	Unclassifiable
**#78**	POS	NEG	Single sample. PCR neg (serum& urine), PRNT ind	Unclassifiable
**#79**	POS	NEG	Single sample. PRNT ind. PCR neg (serum)	Unclassifiable
**#80–#91**	POS	NEG	Single sample. PRNT ind	Unclassifiable
**#92–#99**	POS	NEG	Single sample. No more results	Unclassifiable
**#100**	POS	NEG	Single sample. PCR neg (serum), PRNT pos. DENV (IgM pos, NS1 Ag neg)	Unclassifiable
**#101**	POS	NEG	Single sample. PRNT ind. DENV (IgM pos)	Unclassifiable

**Table 5:** Performance characteristics of IIF and ELISA after the final classification of samples.

		Final Classification	
		Positive (126)	Negative (102)
**Indirect Immunofluorescence**	Positive	122	28
	Negative	4	74
**ELISA**	Positive	83	0
	Negative	43	102
